# Validation of pharyngeal findings on sleep nasopharyngoscopy in children with snoring/sleep disordered breathing

**DOI:** 10.1186/1916-0216-43-13

**Published:** 2014-06-11

**Authors:** Maleka Ramji, Vincent L Biron, Caroline C Jeffery, David WJ Côté, Hamdy El-Hakim

**Affiliations:** 1 Division of Pediatric Surgery, The Stollery Children’s Hospital, University of Alberta Hospitals, 2C3.57 Walter MacKenzie Centre, Edmonton, T6G 2R7 AB Canada; 2 Department of Surgery, The Stollery Children’s Hospital, University of Alberta Hospitals, Edmonton, AB, Canada; 3 Division of Otolaryngology, The Stollery Children’s Hospital, University of Alberta Hospitals, Edmonton, AB, Canada; 4 Department of Pediatrics, The Stollery Children’s Hospital, University of Alberta Hospitals, Edmonton, AB, Canada

**Keywords:** Sleep apnea, Children, Endoscopy, Propofol, Agreement, Diagnosis, Adenotonsillectomy

## Abstract

**Objective:**

To validate the pharyngeal findings in sleep nasopharyngoscopy (SNP) of children with snoring - sleep disordered breathing (S-SDB).

**Design:**

Prospective agreement diagnostic study on retrospective data.

**Methods:**

We conducted an inter-and intra-rater agreement study on video documentations of SNP performed on children (non-syndromic, complex, or operated upon) who presented with S-SDB. The videos featured various pharyngeal findings (normal, collapse, mixed or obstruction). Three ‘non-expert’ raters at various stages in their otolaryngological careers rated the videos independently, and on two separate occasions following an instructional session. We calculated both weighted and non-weighted linear kappa.

**Results:**

Each independent observer rated sixty-one videos (2 weeks apart). Intra-observer agreement was 0.64 ± 0.08 (95% CI 0.48-0.81), 0.74 ± 0.07 (95% CI 0.60-0.88), 0.59 ± 0.08 (95% CI 0.43-0.74), for raters 1, two and three. Weighted kappa was 0.6 ± 0.1 (95% CI 0.41-0.79), 0.8 ± 0.06 (95% CI 0.7-0.92), 0.7 ± 0.07 (95% CI 0.57-0.83), respectively. Inter-rater agreements between raters one and two, two and three, three and four were 0.83 ± 0.06 (95% CI 0.71-0.95), 0.52 ± 0.08 (95% CI 0.36-0.70), and 0.53 ± 0.08 (95% CI 0.37-0.69), respectively. Weighted kappa was 0.83 ± 0.073 (95% CI 0.69-0.98), 0.68 ± 0.06 (95% CI 0.56-0.79), and 0.64 ± 0.07 (95% CI 0.49-0.78), respectively.

**Conclusions:**

This is the first validation of pharyngeal findings on SNP in children. It is based on a four types’ classification. Overall reproducibility amongst the three raters and their agreement was moderate to good. Further work should be phase four trials investigating the impact on outcome.

## Introduction

Sleep disordered breathing (SDB) is commonly diagnosed in the pediatric population. It is defined as a disorder of breathing characterized by ‘prolonged partial upper airway obstruction and/or intermittent complete obstruction that disrupts normal ventilation during sleep’ [1]. Obstructive sleep apnea (OSA), the most severe category of SDB, affects approximately 1-4% of all children. If left untreated, OSA can lead to significant impairment in quality of life and physical health sequelae [2].

Tonsillar and adenoid hypertrophy have been recognized as commonest obstructive pathology that lead to pediatric OSA, and as a result adenotonsillectomy (T&A) is recommended as a first line surgical treatment. However, complete resolution of symptoms after this intervention is infrequent, as estimated in a recent meta-analysis (66.3%). Furthermore, residual disease has been noted to be more prevalent in obese children [3-5].

Sleep endoscopy or nasopharyngoscopy (SNP) has recently gained interest among pediatric otolaryngologists for its potential to identify anatomical sites amenable to surgical correction. *Durr et al.* evaluated post-operative T&A patients with residual symptoms, using drug-induced sleep endoscopy. As expected, their study revealed multi-level obstruction along the upper airway related to the tongue base, adenoid re-growth and inferior turbinate hypertrophy [6]. Although this study used a standardized, site specific scale to assess SNP findings, it has not been accepted nor validated in children. Myatt and Beckenham were the first to describe a specific scoring for levels of obstruction using SNP. They described 4 levels of obstruction, namely the velopharynx, tonsils/lateral pharyngeal wall, tongue base and supraglottis [7].

In the adult literature, two studies conducted by the same research team, evaluated test-retest reliability and inter-rater reliability when using SNP in patients with SDB. The authors found that their intra-rater and inter-rater agreement, on both studies ranged from moderate to substantial. However, their population was heterogeneous with a predominance of abnormal findings, and the assessors were both experienced sleep surgeons [8,9].

The aims of our study are (a) to introduce a specific scoring system to evaluate the pharyngeal findings of SNP in patients with SDB, (b) to validate this scoring system using three raters of unequal experience, who are not experts on SNP.

## Material and methods

We conducted an intra- and inter-rater agreement study at a tertiary referral center (the Stollery Children’s Hospital, Edmonton, Alberta, Canada) after obtaining approval by the institutional Health Research Ethics Board (Pro00024340). Digital videos of patients undergoing SNP were accessed for this study. The videos had been recorded in a standardized manner, employing a pediatric flexible bronchoscope and collected using a digital 3-chip camera and integrated digital data archiving by the senior author of all patients undergoing SNP since August 2003.

SNP was used in all children (<18 years of age) who presented with new or recurrent symptoms of S-SDB, and were potential candidates for surgical management or required exclusion of that option prior to minimally invasive ventilation. The children presented with persistent snoring (witnessed by their parents or care givers for ≥12 months on a nightly basis). Children also presented with other nocturnal and diurnal symptoms. The senior author’s practice uses a modified version of the Pediatric Sleep Questionnaire [10]. In addition to the standard items, we inquired about risk factors of S-SDB perinatally, atopy and other lung conditions, prior surgery, body weight, developmental history, neuropsychiatric conditions, esophagitis, aspiration, and smoking habits of caregivers. All children were subjected to overnight pulse oximetry. The results are graded according to *Nixon et al.*[11]. A full polysomnography was reserved in this practice for syndromic children or those with complex medical history, patients whose diagnosis was in doubt, or whose symptoms were not in concordance with sleep oximetry results.

All SNP were performed with a uniform sedation protocol in the operating room, using Remifentanyl 2–2.5 mcg/ml and infusion rates of Propofol varying from 200–350 mcg/kg/min titrated for response to stimulation. The patients were kept spontaneously breathing throughout the assessment. If inhalational induction had been utilized, the endoscopy was done only when end tidal sevofluorane was zero. The nasal mucosa was topicalized with 1% lignocaine (to a maximum of 3 mg/kg body weight). A flexible bronchoscope was used to assess the airway systematically, from the nose to the larynx.

Sixty-one videos were chosen for the study by the senior author. Allowing for an earlier period of growing experience, and utilization of analogue (non-digital) capture equipment, the records of the first 4 years were avoided. The inclusion criteria were: (1) non-edited, high quality recordings (2) representative of one of encountered types of pharyngeal findings (normal [0], obstruction [1], collapse [2], mixed [3], (3) performed in non-previously operated patients). Aside from ensuring a non skewed proportion of the four types, a random folder of digital videos was chosen from the 6^th^ year (2010), and the videos were included consecutively. There were ultimately thirteen obstruction videos, thirteen collapse, nineteen mixed, and sixteen normal. None of the children whose videos were included were syndromic or complex.

Three “non-expert” raters, at various stages in their otolaryngology career, who had not been involved in the inception of this scoring system, nor do they perform SNP routinely were recruited. Throughout their training they were exposed to SNP for a total of a three-month period. At the time of the experiment, two were starting third and fourth year in residency respectively, and the third had been in staff position for one year after finishing a year of post-graduate clinical fellowship training (head and neck reconstructive and esthetic surgery). The scoring process was explained during an hour-long instructional session. They were blinded to the identity of children, their demographics, clinical details and eventual or prior management. Videos were compiled, coded, and organized into two software presentation documents whose linked videos were de-identified. This process was done by one of us, who was the only one who kept the code to the videos. Each document contained the same videos, but in two different random orders. Each rater was given the 1st set of videos and asked to score them independently. Two weeks later, the rater was given the second document and asked to score the videos.

### Scoring

Each video represented either a normal pharynx, a collapse of the pharyngeal walls affecting over 50% of the cross sectional area during inspiration, an obstruction of the pharynx affecting over 50% of the cross sectional area at both phases of respiration, or a mixed (collapse and obstruction) presentation (Table 1). The raters were required to decide if the type was present or absent. They were not required to rate any nasal, nasopharyngeal or velopharyngeal findings (i.e. started scoring findings from seeing the oro-pharyngeal tonsils, downwards). The main objective was to rate the oro- and hypopharynx as these were the regions deemed most likely affected by pharmacologically-induced sleep.

**Table 1:** Summary of scoring instructions

***Description***	***Finding***
No collapse or obstruction	Normal
>50% compromise throughout the respiratory cycle. No pharyngeal wall movement	Obstruction
>50% compromise throughout the cycle & visible paradoxical movement of the pharyngeal wall during *inspiration*	Mixed
Paradoxical movement of pharyngeal wall during *inspiration* or tongue collapse *throughout*.	Collapse
*No* obstruction	

### Statistics

Kappa statistic was used to measure agreement. Non-weighted kappa was calculated first. We then postulated that since the normal and collapse states do not require pharyngeal surgery (assuming no other variable interferes with the decision), the rater’s scoring should be penalized upon rating them as obstruction or mixed states. As such linear weighted kappa calculation was based on unequal imputed distance (doubled) between the first two categories and the third and fourth. The kappa values, standard errors, maximum possible kappa, proportions and 95% confidence intervals were provided [12].

## Results

A total of 61 videos were analyzed. There were thirteen obstruction videos, thirteen collapse, nineteen mixed, and sixteen normal. The mean duration was 52 ± 26.99 seconds (range 15–180 seconds). The mean age was 7.43 ± 2.37 (4.3-6.25) years. Thirty-one were males. The mean BMI for age and sex was 20.9 ± 2.5 kg/m [2]. Median pulse oximetry grade was 1.

Three raters scored the videos as described in the Materials and methods section. The intra-rater agreement ranged from moderate to good for non-weighted kappa values (Table 2). The values were 0.64 ± 0.078 for rater 1, 0.73 ± 0.071 for rater 2, and 0.58 ± 0.0776 for rater 3. The 95% confidence interval (CI) spanned two categories of agreement (i.e. moderate to good or good to very good). The proportions of agreement were 0.77, 0.82, and 0.69 for raters 1, 2, and 3 respectively. They were all higher than expected for chance alone.

**Table 2:** Intra-rater agreement

	***Rater 1***	***Rater 2***	***Rater 3***
***Unweighted K***			
Observed	0.64	0.74	0.589
Maximum Possible	0.87	0.83	0.87
Standard Error	0.08	0.07	0.08
95% CI	0.49-0.80	0.60-0.88	0.43-0.74
***Linear Weighted K***			
Observed	0.60	0.81	0.67
Maximum Possible	0.80	0.84	0.88
Standard Error	1.00	0.06	0.07
95% CI	0.41-0.79	0.70-0.92	0.57-0.83
***Proportions of Agreement***			
Observed	0.77	0.82	0.69
Maximum Possible	0.92	0.89	0.90
Chance expected	0.36	0.32	0.25
95% CI	0.64-0.86	0.70-0.90	0.56-0.80

Linear weighted kappa values were slightly higher, and also ranged from moderate to good. The values were 0.60 ± 0.1 for rater 1, 0.80 ± 0.06 for rater 2, and 0.7 ± 0.07 for rater 3, and their 95% CI lower limit were moderate.

Table 3 displays inter-rater agreement. Raters one and two agreement was the highest (very good). The non-weighted kappa was 0.85 ± 0.0569, the weighted value was 0.83 ± 0.07, and the observed proportion of agreement was 0.9 (Table 2). The next two sets of agreements were moderate on non-weighted kappa (0.53 ± 0.08, and 0.53 ± 0.08 for raters 2 & 3, and 1 & 3 respectively). Both improved to good on calculating linear weighted kappa (0.68 ± 0.07, and 0.64 ± 0.07 for raters 2 & 3, and 1 & 3 respectively). Both observed proportions of agreement were similar (0.66) and above that expected by chance.

**Table 3:** Inter-rater agreement for three observers

	**Rater 1 & 2**	**Rater 2 & 3**	**Rater 1 & 3**
***Unweighted K***			
Observed	0.85	0.53	0.53
Maximum Possible	0.95	0.69	0.69
Standard Error	0.057	0.08	0.08
95% CI	0.74-0.96	0.37-0.68	0.38-0.68
***Linear Weighted K***			
Observed	0.83	0.68	0.64
Standard Error	0.07	0.06	0.07
Maximum Possible	0.90	0.74	0.72
95% CI	0.69-0.98	0.56-0.79	0.49-0.78
***Proportions of Agreement***			
Observed	0.90	0.66	0.66
Maximum Possible	0.97	0.77	0.77
Chance expected	0.35	0.27	0.27
95% CI	0.79-0.96	0.52-0.77	0.52-0.77

## Discussion

According to accepted categories of kappa values intra- and inter-rater agreements in this study are good [13]. Generally speaking, by rejecting the null hypothesis (k is not zero, and above 0.5) we are assured that the agreement achieved is above chance, but its interpretation to individual situations will vary [14].

In this study, we have achieved these results by non-expert raters in order to demonstrate that the method is easy to learn, and reproducible. A conscious attempt was made to test the most contentious of issues related to SNP: the oro- and hypopharyngeal findings. Although the same technique is used on a daily basis for diagnosing and managing dynamic laryngeal conditions, concerns exist that pharmacologically induced sleep would distort the findings. Such issues are not valid for the nose and nasopharynx, where changes might only be affected by posture and use of decongestants [15]. We also sought to cater for one of the most important proposed functions of SNP; seeking surgical targets and avoiding unnecessary operations. By calculating linear weighted kappa, the ratings incurred a heavier penalty upon disagreement where surgery may be useful (normal/collapse versus obstruction/mixed) and not just mis-classification of the mutually exclusive types of finding.

Although the videos used were not recorded for the purpose of the study, the conditions of the endoscopies were standardized, and the design of the experiment was conceived prospectively. Another caveat that we circumvented in this study is spectrum bias [16]. In contrast to other studies (*Durr et al., Truong et al.*) we have neither included children who were operated upon before nor complex or syndromic children, and a broad range of findings were included [6,17]. This lends more credibility to the findings, and less room for learning effect and chance agreement.

All the endoscopies were performed while the patients were breathing spontaneously under the same intravenous agents, although it is conceivable that in a full prospective experiment some endoscopies might have been excluded due to protocol deviations. There is some debate, however, regarding the ideal pharmacological agent that would achieve the closest status to physiologic sleep. Current literature suggests that a clinical target of loss of responsiveness can be used to achieve airway conditions that mimic findings seen in normal sleep using either propofol, or midazolam infusion [18]. Further, we have evidence from the literature in favor of propofol based on comparable polysomnography findings to those of physiological sleep [19], its effect on the genioglossus muscle, critical closing pressure of the pharynx, and its titratable effects [20-28]. The caveat is that these citations are all from adult literature.

One plausible criticism is the conspicuous absence of PSG, the reference standard for the diagnosis. We would argue that we have used a validated score based on pulse oximetry, and the patients were screened with a standard questionnaire. Further, the agreement study in its own right would not have been affected, and correlating the findings to PSG was not our set objective.

There are two further limitations to this work. These are namely the absence of test-re-test reliability, and site specific testing. As to the former, there are ethical considerations that probably would have made that step impossible. These relate to concerns regarding consequences of repeated general anesthesia on the health of children, despite the evidence being controversial [29]. With respect to individual sites (e.g. scoring for laryngomalacia, lingual tonsil enlargement, lateral versus circumferential collapse), this work did not aim beyond testing the agreement on discriminating normal, collapse and obstruction. The latter aspect is an important step towards evaluating this diagnostic tool [30]. Ultimately, after external validation the community should put to the test whether surgery directed by SNP would achieve better results that traditional planning. This could also unravel the reasons for the current success figures of adenotonsillectomy in the treatment of SDB.

The findings of this study have implications for the management of SDB in children. To date, no such validation exercise has been done for assessment of SNP in pediatric patients. *Kezirian et al.* published two prospective studies assessing test-retest reliability and inter-rater reliability of DISE in adults with SDB. They found both test-retest and inter-rater reliability to be moderate to good [13,31]. Our work supports the notion that SNP in general is a promising tool in SDB.

We have published, and unpublished data that demonstrate, in one cross-sectional [32] and three case-controlled studies [27,33,34] that SNP findings in children presenting with SDB, and particular risk groups are unique and different from comparison groups. Using comparisons of the proportions of collapse, obstruction and mixed findings the comparison groups demonstrated consistently a predominance of mixed and obstructive findings over that of collapse, whereas the high-risk groups demonstrated more collapse and mixed groups. These may be viewed as phase one trials demonstrating that the findings in of SNP are distinct in high risk groups of SDB [30].

A final point that would emphasize the potential impact of this practice on changing management decisions comes from the difference in SNP findings and those of traditional clinical examination in the awake child. Upon conducting an agreement analysis on obstructive and non-obstructive finings in our first 248 children, the k was 0.44 (95% CI 0.33-0.55). The clinic findings missed 58 obstructions, and misdiagnosed 13 non-obstructions (considering the SNP as the reference standard). Ostensibly, many useful surgeries would have been missed, and some unnecessary operations performed.

## Conclusion

We have demonstrated a moderate to good agreement on a proposed scoring of the pharyngeal findings of SNP in children with snoring/SDB. External validation and phase four trials are recommended for future work.

## Competing interests

The authors declare that they have no competing interests.

## Authors’ contributions

MR: put the original protocol, submitted the ethics approval application, helped in creating the final format of the digitized videos, anonymized, and created the random sequence for them. She collected the scoring sheets. She wrote the original draft of the manuscript, and incorporated the revisions. VB: scored the videos, assisted in critical appraisal of design and statistical analysis, revised the manuscript. CCJ: scored the videos, assisted in critical appraisal of design, and revised the manuscript. DWC: scored the videos, assisted in critical appraisal of design and statistical analysis, revised the manuscript. HE: Conceived the idea and put the original protocol, Reviewed and selected the digitized videos. He collated the data from the scoring sheets and performed the statistical analysis and interpretation of data. He revised the original draft of the manuscript, provided the final version. All authors read and approved the final manuscript.
